# Effects of Feeding either Red or White Grape Marc on Milk Production and Methane Emissions from Early-Lactation Dairy Cows

**DOI:** 10.3390/ani10060976

**Published:** 2020-06-04

**Authors:** Peter J. Moate, Joe L. Jacobs, Josh L. Hixson, Matthew H. Deighton, Murray C. Hannah, Greg L. Morris, Brigid E. Ribaux, William J. Wales, S. Richard O. Williams

**Affiliations:** 1 Agriculture Victoria Research, Ellinbank VIC 3821, Australia; joe.jacobs@agriculture.vic.gov.au (J.L.J.); matthew.deighton@cropmark.co.nz (M.H.D.); murray.hannah@agriculture.vic.gov.au (M.C.H.); greg.l.morris@agriculture.vic.gov.au (G.L.M.); brigid.ribaux@agriculture.vic.gov.au (B.E.R.); bill.wales@agriculture.vic.gov.au (W.J.W.); richard.williams@agriculture.vic.gov.au (S.R.O.W.); 2 Centre for Agricultural Innovation, School of Agriculture and Food, Faculty of Veterinary and Agricultural Sciences, The University of Melbourne, Victoria 3010, Australia; 3 The Australian Wine Research Institute, P.O. Box 197, Glen Osmond, Adelaide 5064, Australia; josh.hixson@awri.com.au; 4 Cropmark, 49 Manion Road, Rolleston 7677, New Zealand

**Keywords:** enteric methane, cattle, sulphur hexafluoride, milk production

## Abstract

**Simple Summary:**

Grape marc comprises the skins, seeds and stems of grapes remaining after grapes are pressed to make wine. Globally, about nine million tonnes of grape marc are produced annually. However, little is known about the comparative nutritional value of grape marc from red and white grapes and their effects on milk production and methane emissions when fed to dairy cows. Our experiment assessed the potential role of grape marc as a feed source for the grazing based, Australian dairy industry. We fed diets based on harvested perennial ryegrass to lactating dairy cows and compared milk production and methane emissions when grape marc from either red or white grapes was substituted for some of the perennial ryegrass. Diets containing grape marc from either red or white grapes equally decreased milk yields by approximately 10% and methane emissions by 15%. When fed to dairy cows, grape marc reduces methane emissions but at the cost of decreased milk production. The effects on methane emissions were mainly mediated by the high concentrations of lignin and fat in grape marc while decreased milk production was due to decreased intake of metabolizable energy.

**Abstract:**

Globally, annual production of grape marc (GM), the residue of skins, seeds and stems remaining after making wine, has been estimated to be approximately nine million tonnes. No previous studies have compared effects on milk production and methane emissions when GM from either red or white grapes was fed to dairy cows. This experiment examines the effects of partial replacement of a perennial ryegrass (*Lolium perenne* L.) based diet with GM from either red or white grapes on yield and composition of milk and methane emissions. Thirty-two Holstein dairy cows in early lactation were offered either a control diet containing 15.0 kg dry matter (DM) of freshly harvested perennial ryegrass and 5.2 kg of a concentrate mix, or a diet similar to the control diet but with 5 kg DM of ryegrass replaced with 5 kg DM of GM from red grapes (RGM), or a diet similar to the RGM diet except the GM was from white grapes (WGM). Individual cow feed intakes, milk yields, and methane emissions were measured. Both diets containing GM decreased milk yields by approximately 10% and methane emissions by 15%. When fed to dairy cows, GM reduces methane emissions but at the cost of decreased milk production.

## 1. Introduction

The dairy industry provides high-quality food to populations around the world, but has a substantial carbon footprint [1]. Recent efforts to reduce the carbon footprint of the dairy industry have focused on the use of agricultural by-products as dietary supplements for dairy cows (e.g., [2,3]). The rationale behind this is that by-products are generally inexpensive and production of by-products can, from a life-cycle analysis perspective, be considered free of greenhouse gas emissions as any greenhouse gas released during the production of the by-product can be attributed to the primary product [4]. Considerable research has been conducted into by-products rich in fat and forages rich in condensed tannins as these have been shown to reduce enteric methane production when fed to ruminants [3,5,6]. By-products may sometimes have elevated concentrations of specific long-chain fatty acids such as linolenic acid, regarded as being beneficial for human health, and when these by-products are fed to dairy cows, the resulting milk fat may become enriched in these fatty acids [3,7]. In addition, by-products of agricultural industries are generally not considered suitable as a foodstuff for humans, but in a world increasingly food insecure, they can be used as a feed supplement for animals [8], and can therefore indirectly contribute to the nutrition of humans.

In Australia, dairying is mainly based on dairy cows grazing pasture with various other feeds, especially cereal grains, fed as supplements to the pasture [9,10]. However, during dry summer months and during years when droughts occur, pasture availability becomes limiting and cereal grain feed supplements may become prohibitively expensive or unavailable. Under these circumstances, dairy farmers may be faced with the decision to either underfeed their cows or to offer them a by-product such as grape marc.

Grape marc is the skins, seeds and stems remaining after grapes (*Vitis vinifera* L) have been pressed to make wine. Grape marc constitutes approximately 25% of grapes used in wine production [11], and as annual global wine production is approximately 27 M tonnes, it can be estimated that global production of grape marc is approximately 9 M tonnes per annum, with approximately 374,000 tonnes per annum of grape marc (~60% red grape marc from red grapes and ~40% white grape marc from white grapes) produced in Australia [11,12]. Grape marc generally contains a high concentration of lignin, which is not fermented in the rumen or lower gut, and therefore grape marc generally has a low concentration of metabolizable energy and is regarded as having low to moderate nutritive value [13,14]. Grape marc may also contain high concentrations of both fat (5.2 to 185 g/kg DM) and condensed tannins (6.9 to 139 g/kg DM); both of which are known to inhibit enteric methane production [1,15,16]. Indeed, when dairy cows were fed grape marc from red grapes, methane emissions (g/d) and methane yield (g/kg dry matter intake, DMI) were reduced by approximately 20% [14]. The concentrations of both fat and tannins in grape marc differ considerably depending upon many factors, including variety and colour of grape and the different pressing processes associated with making red and white wines [15,16,17]. An in vitro evaluation of the methane mitigation potential of 20 grape marcs found that grape marc from red grapes produced 13% less methane than grape marc from white grapes [17], but we have been unable to find any published studies undertaken in vivo that support this finding. A number of studies have examined the effects of feeding various grape marcs to dairy cows on their milk production and milk composition [13,18,19], but we are unaware of any studies that have directly compared the effects of feeding red or white grape marc to dairy cows on their milk production, milk composition and methane emissions. However, the feeding of diets containing approximately 27% red grape marc to dairy cows substantially reduced yields of milk, energy corrected milk (ECM), milk fat, milk protein and milk lactose [14]. In contrast, when Santos et al. [18] fed a diet containing less than 10% grape marc from purple grapes, or when Ianni et al. [20] fed a diet containing less than 10% dried grape marc from red grapes, both studies reported no effects on yields of milk fat, milk protein or milk lactose of dairy cows. We have been unable to find studies that have reported the effects of grape marc from white grapes on milk production and methane emissions.

The objective of this research is to compare the effects on milk yield, milk composition and methane emissions of dairy cows when the pasture component of their diet was partially replaced by grape marc derived from either red or white grapes. We hypothesized (1) that feeding diets containing approximately 24% (on a DM basis) of red or white grape marc instead of harvested perennial ryegrass (*Lolium perenne* L.) pasture to dairy cows would reduce yields of milk, milk fat and milk protein. In the absence of published in vivo data comparing the effects of feeding either red or white grape marc, we also propose a null hypothesis (2) that cows fed diets containing either red or white grape marc would produce similar yields of milk, milk fat and milk protein; and (3) that when either red or white grape marcs are included in a diet, they would inhibit methane emissions from dairy cows to an equal extent.

## 2. Materials and Methods

The experiment was conducted at the Agriculture Victoria Research, Ellinbank Dairy Centre, Victoria, Australia (38°14′ S, 145°56′ E) and was conducted in accordance with the Australian Code of Practice for the Care and Use of Animals for Scientific Purposes [21]. Animal use was approved by the Animal Ethics Committee of Agriculture Victoria (2013–11, 15 August 2013).

### 2.1. Cows, Diets, Feeding and Management

Three dietary treatments were assigned to 32 lactating, multiparous Holstein Friesian cows (501 ± 51.9 kg bodyweight, 22.1 ± 5.1 days in milk, 28.1 ± 5.58 milk kg/d, 3.4 ± 0.50 years of age, mean ± standard deviation) at random, subject to treatment group balanced for bodyweight, days in milk and age, according to the method of Harville [22], using procedure COVDESIGN in GenStat 18 statistical software (VSN International Ltd., Hemel Hempstead, UK). The 3 dietary treatments were: (1) a control diet (CON) in which cows were individually offered 15.0 kg DM daily of freshly-harvested, single-chop, perennial ryegrass pasture; (2) a red grape marc diet (RGM) in which cows were individually offered 5.0 kg DM of red grape marc and 10.0 kg DM of freshly cut perennial ryegrass pasture; (3) white grape marc diet (WGM) in which cows were individually offered 5.0 kg DM of white grape marc and 10.0 kg DM of freshly cut perennial ryegrass pasture. The pasture was harvested twice daily (06:00 and 14:00 h) at a pre-flowering stage and a pasture mass of approximately 2500 kg/ha using a front-mount mower (Novacat 356F; Pottinger, Grieskirchen, Austria), a tractor (Arion 530; Claas, Harsewinkel, Germany) and a loader wagon (Quantum 3500P; Claas, Harsewinkel, Germany) was used. All dietary treatments also included 3.0 kg DM of cracked corn (*Zea mays* L.) grain, 2.0 kg DM of cold pressed canola (*Brassica napus* L.) meal and 0.2 kg DM of minerals (calcium 134 g/kg DM, magnesium 110 g/kg DM, phosphorus 60 g/kg DM, zinc 6.4 g/kg DM, manganese 1.8 g/kg DM, copper 1.2 g/kg DM, iodine 80 mg/kg DM, cobalt 100 mg/kg DM and selenium 24 mg/kg DM). Cows were fed in individual stalls in a well-ventilated animal house [23]. The diets were offered to cows in two equal portions during two 4-h periods following the morning milking at 07:30 h and the afternoon milking at 15:30 h. The concentrate portion of the diet was offered to the cows first, and they were allowed 30 min to consume their concentrate, at which time, any remaining concentrate was removed and weighed. The grape marc portion of the diet was then offered to the cows, and cows were allowed 1 h to consume the grape marc. The quantum (5 kg DM) of grape marc offered to cows was chosen as this is an amount commonly offered to dairy cows on commercial farms in Australia and we successfully fed a similar amount to dairy cows in a previous experiment [14]. Cows offered the CON dietary treatment received the pasture portion of their diet after they had eaten their concentrate, while cows offered the RGM and WGM treatments received the pasture portion of their diet after the grape marc. The pasture was not machine-chopped in order to maintain its physical and chemical attributes to be similar to grazed pasture. The diets were intentionally not fed as a total mixed ration (TMR), as these feeding treatments and regimen of feeding the concentrate and grape marc portions of the diet separately before the pasture portion of the diet were chosen to mimic the diets and feeding regimens that typically occur on Australian dairy farms. Furthermore, the dietary regimens were chosen as described because it is not practical to formulate a pasture based TMR of fixed chemical composition and balanced for major nutrients as pasture can change substantially from day to day in terms of DM concentration and nutrient concentration. On this point, we intentionally did not try to make the diets isoenergetic and isonitrogenous as the disparate chemical compositions of the pasture and grape marcs would have made this impossible without additional dietary components, and hence confounding factors, thereby making the experiment not relevant in terms of how grape marc is fed as a supplement on Australian dairy farms.

The red and white grape marcs (Tarac Technologies Pty. Ltd., Nuriootpa, South Australia, Australia) both contained skins and seeds, and had undergone a crimping process. Crimping involves screening to remove stalks then rolling to crush the seeds; processes that improve digestibility and energy availability. Both grape marcs were ensiled in large plastic bags from which air had been evacuated by means of a vacuum pump, and the bags were then sealed so as to be airtight. Each bag contained sufficient grape marc for only one day’s consumption by the dairy cows so as to limit the potential of aerobic spoilage of the grape marcs when the bags were opened.

The CON treatment was assigned to 12 cows, while the RGM and WGM treatments each had 10 cows. Following a 5-d covariate period during which milk yield and composition were measured, cows were transitioned to their diets on days 1 to 4, allowed to adapt to the diets on days 5 to 14, and measured on their assigned diets during days 15 to 28.

### 2.2. Measurements

Samples of concentrates offered were collected once each week for DM determination by drying in a forced-draft oven at 105 °C for 48 h. Samples of pasture and grape marcs offered at each feeding were collected daily and DM determined for each feed by drying in a forced-draft oven at 105 °C for 48 h.

Representative samples of cracked corn grain and cold-pressed canola offered were collected each feeding, frozen and bulked over the duration of the experiment. Representative samples of pasture and of grape marcs were collected daily, frozen and bulked weekly. Frozen samples were subsequently freeze-dried, ground to pass through a 0.5 mm sieve and then chemically analysed (Dairy-One Forage Laboratory, Ithaca, NY, USA) for crude protein, neutral detergent fibre and crude fat according to the methods of Dairy One [24]. Gross energy was calculated using an equation derived from Klop et al. [25], and metabolizable energy (ME) was calculated according to the National Research Council [26]. Potentially fermentable organic matter (PFOM, g/kg DM) for each diet ingredient “i” was calculated as: 1000 − ((Ash) + (Ki × Crude fat) + Lignin), where Ki is a factor for each dietary feed ingredient, and represents the proportion of crude fat in feed ingredient “i” that is composed of non-fermentable fatty acids. For most fats of plant origin, Ki has a value of 0.9 while for fats from temperate grass species, including perennial ryegrass, Ki has a value of 0.65 [27,28].

Freeze-dried and ground samples of red and white grape marc were analysed for tannin as described by Hixson et al. [29] and all quoted values are a mean of triplicate analyses. Briefly, total condensed tannin (CT) was determined as the sum of all flavan-3-ol subunits resulting from depolymerization in the presence of phloroglucinol (phloroglucinolysis). Late eluting material (LEM), a measure of oxidized cross-linking between flavan-3-ol subunits, was quantified in epicatechin equivalences. Composition variables were determined from molar subunit ratios: mean degree of polymerization (mDP) from the ratio of total to terminal subunits; the *cis*/*trans* ratio from 2,3-*cis* based to 2,3-*trans* based subunits; percentage galloylation (%Gall) from the occurrence of epicatechin-O-3-gallate terminal and extension subunits; and percentage of prodelphinidin subunits (%PD) from the occurrence of epigallocatechin terminal and extension subunits. Tannin analysed by the acetone-butanol-HCl method (TAB-HCl) and highly bound tannin (HBT) were determined from depolymerization and colorimetric quantification at 550 nm against a grape-derived tannin standard using either whole marc fibres (TAB-HCl) or freeze-dried marc fibres remaining post-phloroglucinolysis (HBT). Water extractable tannin (WET) was determined from an aqueous extract (24 h, room temperature) and quantified using methyl cellulose precipitation against a catechin standard.

Milk yields of individual cows were measured morning and afternoon for the duration of the experiment using a DeLaval ALPRO milk metering system (MM25; DeLaval International, Tumba, Sweden). Milk samples for composition analysis were collected from Tuesday afternoon milking to Friday morning milking (inclusive) during the covariate period, and from Tuesday afternoon milking to Friday morning milking (inclusively) during the measurement period. Fat, protein and lactose in milk were measured by means of a near-infrared milk analyser (model 2000, Bentley Instruments, Chaska, MN, USA). Somatic cells were counted by a Fossomatic SC300 cell counter. Energy-corrected milk, standardized to 4.0% fat and 3.3% protein, was calculated using the following formula [30].

ECM (kg/d) = (milk yield (kg) × (376 × fat% + 209 × protein% + 948))/3138(1)

All milk from the afternoon milking of day 25 and morning milking of day 26 was collected and subsampled. Fatty acid proportions were determined according to Moate et al. [3] from a composite sample of afternoon milk and morning milk proportional to the volume of each milking, with the sample stored at −18 °C until analysis.

Individual cows were weighed after the morning milkings on days 6 to 8 and on days 32 to 34 to enable estimation of average daily bodyweight change.

### 2.3. Methane Emissions

Methane emissions (g/d) from each cow were measured from day 24 to 29 using a SF_6_ tracer technique similar to that described by Deighton et al. [31]. In this experiment, the permeation tubes were manufactured in-house in September 2013 and were filled with about 2.4 g of SF_6_. The release rate of SF_6_ was determined by storing the permeation tubes in an incubator (Heratherm Advanced Protocol; Thermo Fisher Scientific, Waltham, MA, USA) set at 39.0 °C and weighing the tubes every second day over a 14 day period immediately prior to use, with the release rate determined by linear regression. The release rate of SF_6_ was 5.2 ± 0.04 mg/d (average ± standard deviation) and ranged from 5.15 to 5.29 mg/d. The permeation tubes containing SF_6_ were placed in gelatine capsules (Size #10, Torpac, Fairfield, NJ, USA) then inserted into the rumen of the cows per os 6 days before the first cow-breath samples were collected. Stainless steel canisters of 800 mL and a sampling rate of ~0.2 mL/min were used to continuously sample eructated gases. Eight sentinel canisters [23] were used to sample background gases indoors (~0.8 mL/min), 4 canisters were used, one on each fence of the loafing area to sample background gases outdoors (~0.3 mL/min), and orifice plates (Lenox Laser, Glen Arm, MD, USA) were used to control the gas sampling rate [31]. Canisters were exchanged once per day. Overall concentrations of background gases for individual cows were the time-weighted average of their indoor and the group outdoor concentrations. Two of the 160 cow samples were lost, while all 60 background samples were collected.

The vacuum of evacuated canisters was measured using a digital gauge (XP2i-DP, Crystal Engineering, San Luis Obispo, CA, USA) and recorded. When the canisters were used to collect breath samples, the vacuum of the individual canisters was again measured and recorded. Ultra-high-purity N_2_ (99.999%) was then added to each canister to achieve a vacuum of ~10 kPa with the actual vacuum achieved being measured and recorded.

Gas samples were analysed by gas chromatography [23]. After analysis, the dilution by N_2_ was reversed mathematically to account for the physical dilution of the samples with N_2_ gas prior to analysis (Equation (2)):

(2) $$\left[G_{S}]=\frac{101-\tau_{f}}{\tau_{e}-\tau_{s}}\times [G_{A}\right]$$

where [*G_S_*] is the calculated concentration (ppm for methane, ppt for SF_6_) of the gas as sampled; 101 is the average atmospheric pressure (kPa); *τ_f_* (kPa) is the final vacuum in the canister after the addition of nitrogen; *τ_s_* (kPa) is the vacuum in the canister after the sample is collected; *τ_e_* (kPa) is the vacuum in the evacuated canister before use; and [*G_A_*] (ppm for methane, ppt for SF_6_) is the gas concentration in the sample presented to the gas chromatograph. Methane emissions for each day were calculated using Equation (2) from Williams et al. [23].

### 2.4. Ruminal Fermentation and Protozoa Counts

A sample (~400 mL) of ruminal fluid was collected from each cow on day 29 using an oro-ruminal sampling tube similar to the one described by Geishauser [32] and a vacuum pump [14]. The pH of the ruminal fluid was immediately measured using a Mettler-Toledo FiveGo FG2 pH meter (Schwerzenbach, Switzerland).

Ciliate protozoa were counted from a 0.5 mL sub-sample of ruminal fluid transferred to a 12 mL plastic vial, diluted with 4.5 mL of a stain solution containing 10% formalin, 0.9% saline and 0.06% methyl green, then stored at ambient conditions [33] Counting was done in a modified Fuchs-Rosenthal counting chamber using a microscope (Leitz Laborlux S; Leica Microsystems Pty Ltd., North Ryde, NSW, Australia).

Volatile fatty acids (VFAs) were measured from a 5 mL sample of ruminal fluid to which two drops of concentrated (18.4 M) H_2_SO_4_ were added immediately before storage at −18 °C. Subsequent analysis was by capillary gas chromatography according to Supelco Bulletin 749D. D-lactate in ruminal fluid was measured by a Boehringer Mannhein kit (Cat No. 11 112 821 035) on an Olympus AU400 autoanalyzer after deproteinization with perchloric acid. Ammonia-N concentration of ruminal fluid samples was determined from a 0.5 mL sample as described by Moate et al. [34].

### 2.5. Statistical Analyses

Primary data (AM and PM milk yields, AM and PM milk component concentrations and cell counts) from the 14-day measurement period were examined graphically using the Lattice package in R software, plotting each parameter over time separately for each animal. Three outlying fat concentration data, 3 protein concentration data and 1 lactose concentration datum were removed from the dataset. Data were then averaged within weeks for each cow, and results used to compute total milk yield (PM plus AM milk yield), milk-component yields (sum of PM and AM component yields), milk-component concentrations (milk-component yields divided by total milk yield) and daily somatic-cell count (weighted by PM and AM milk yields).

Cow by week data were analysed by split-plot analysis of covariance (ANCOVA) with treatment structure as diet by week and blocking structure as cow split for week using GenStat 18 statistical software. The covariate-period measurement of the corresponding dependent variable was included as a covariate. In addition, variables used for initial balancing treatment groups, namely, age, days in milk, body weight, 7-day average milk yield and their squared terms were included as covariates. ANOVA distributional assumptions of normality and constant variance were examined graphically using histograms of residuals, residuals versus fitted values and normal quantile plots. The somatic-cell count data were log-transformed.

Methane, ruminal VFA and milk fatty acid were analysed by ANCOVA with treatment structure as diet, and blocking structure as cow, using GenStat 18 statistical software. Variables used for initial balancing treatment groups, namely, age, days in milk, bodyweight, 7-day average milk yield and their squared terms were included as covariates.

Protozoa data were analysed as the original counts using a generalized linear model with Poisson error and log link, allowing for over-dispersion, in GenStat 18. Means were predicted on the scale of the linear predictor, back transformed to counts and scaled by the dilution factor to the number of cells per mL.

## 3. Results

Nutritive characteristics of the main ingredients in the diets are shown in Table 1 and concentrations of tannins and their characteristics are shown in Table 2. Concentration of tannin in the red grape marc was less than in the white grape marc, as determined by CT and TAB-HCl (Table 2), with similar WET. Based on the recorded intake of individual dietary ingredients and their nutritional characteristics, the average concentrations of crude protein, neutral detergent fibre, crude fat, lignin and tannin were calculated and are shown in Table 3. Cows offered each diet had similar total DMI (Table 4). Dry matter intakes of pasture and grape marc were different as per the experimental design, and consequently the grape marc diets provided smaller (*p* = 0.008) intakes of ME and smaller (*p* = 0.001) intakes of crude protein, but greater (*p* = 0.001) intakes of neutral detergent fibre, fat and lignin. The two grape marc diets provided similar (*p* > 0.05) amounts of ME, crude protein, lignin and PFOM, but the RGM diet provided greater (*p* < 0.05) amounts of neutral detergent fibre and fat than the WGM diet.

### 3.1. Milk Production and Milk Fatty Acid

The average milk yield and ECM of cows offered RGM and WGM diets were less (*p* < 0.001) than that from cows offered the CON diet (Table 5). Both the RGM and the WGM diets reduced (*p* = 0.001) production of milk fat, protein and lactose as well as the yield of milk fat plus protein (FP) relative to cows fed the CON diet. There were no differences in the yields of milk, ECM, fat, protein or lactose between cows fed the two types of grape marc. Milk fat concentration was lower (*p* = 0.034) for cows offered the RGM diet compared to the other two diets. Concentrations of milk protein from cows fed diets containing both types of grape marc were lower (*p* = 0.04) than in milk from cows fed the CON diet, but there was no difference (*p* = 0.357) in milk protein concentration of cows fed the RGM and WGM diets. Lactose concentrations as well as somatic cell counts in milk were unaffected (*p* > 0.05) by dietary treatments.

Mean concentrations of milk fatty acids from cows offered the CON, RGM and WGM diets are presented in Table 6. Compared with the CON diet, the two grape marc diets resulted in reduced (*p* < 0.01) concentrations of C6:0, C8:0, C10:0, C10:1, C12:0, C14:0, C14:1, Iso C15:0, Anteiso C15:0, C15:0 and C16:0, as well as total de novo produced fatty acid. However, the two grape marc diets resulted in increased (*p* = 0.001) concentrations of C18:0, C18:1 *trans*-9, C18:1 *trans*-10 and C18:2n 6 *cis* as well as total C18 fatty acid and total polyunsaturated fatty acid compared to the CON diet. Grape marc type was associated with some effects on concentrations of specific fatty acids. Concentrations of C10:1, C14:1, C16:0 and total de novo fatty acids were all lower (*p* < 0.05) in the milk fat of cows fed the RGM diet compared to those in the milk fat of cows fed the WGM diet. Conversely, concentrations of C18:1 *trans*-9, C18:1 *trans*-10, C18:1 *trans*-11, C18:2n6 *cis*, conjugated linoleic acid (CLA), total mono-unsaturated fatty acids and total poly unsaturated fatty acids were greater (*p* < 0.05) in the milk fat of cows fed the RGM diet compared to their concentrations in the milk fat of cows fed the WGM diet.

### 3.2. Methane Emissions

Cows offered the RGM and WGM diets had lower (*p* ≤ 0.001) methane emissions and methane yield (g/kg DMI) than cows offered the CON diet. However, dietary treatment had no effect (*p* = 0.336) on methane yield when expressed as (g/kg PFOM) and no effect (*p* = 0.406) on methane intensity (g/kg ECM) (Table 7). There was no effect (*p* > 0.05) of type of grape marc on any of the methane variables.

### 3.3. Rumen Fermentation

Dietary treatment had no effect (*p* = 0.896) on the pH of ruminal fluid, but cows offered the two grape marc diets had lower (*p* = 0.011) concentrations of ammonia in their ruminal fluid compared to that in cows offered the CON diet (Table 8). Dietary treatment had no effect (*p* = 0.728) on the concentrations of total VFA in ruminal fluid. The RGM diet was associated with smaller percentages (of total VFA) of acetic (*p* = 0.002) and caproic acids (*p* = 0.001) and greater percentages of propionic (*p* = 0.001) and iso-butyric acids (*p* = 0.003) than the WGM diet (Table 8). The acetic to propionic ratio in the ruminal fluid from cows offered the RGM diet was smaller (*p* = 0.001) than the ratios in the ruminal fluids of cows offered the WGM diet. The ruminal fluid of cows offered the RGM diet had greater (*p* < 0.05) numbers of *Entodinium* spp. and total protozoa than the ruminal fluid of cows offered the WGM diet.

## 4. Discussion

The nutritional characteristics of grape marcs vary considerably, depending on many factors including the proportions of grape skins, seeds and stems, and winemaking processes [16]. The chemical nutritive characteristics, including the concentrations of lignin, ash, total digestible nutrients (TDN) and ME, of the red and white grape marcs used in this research were within the ranges previously reported [15,16,17] and numerically similar to each other. However, in this experiment, the concentrations of CT, LEM and WET were all numerically greater in the white grape marc than in the red grape marc. In general, colour per se is not necessarily an overriding factor that determines the tannin concentrations in grape marc, and factors such as grape variety, composition of the grape marc in terms of proportions of skin, seeds and stems and other factors appear to influence the concentrations of various types of tannins in grape marc [16].

Lignin concentration in both grape marcs was in excess of 412 g/kg DM. This constitutes a high lignin concentration as the majority of feeds for cattle have a lignin concentration of 50 g/kg DM or less, and of the 121 feeds listed in the NRC [26] feed dictionary, only five had a lignin concentration greater than 100 g/kg DM (almond hulls 149 g/kg DM; apple pomace 154 g/kg DM, whole cottonseed with lint 129 g/kg DM, and solvent extracted safflower meal 145 g/kg DM). The substantial concentrations of lignin in both grape marcs resulted in lignin concentrations of 132 and 137 in the WGM and RGM diets. Ellis et al. [35] analysed data from 29 published papers and reported on the lignin concentrations in 83 diets for beef cattle and 89 diets for dairy cows. Mean dietary lignin concentrations were 57 ± 1.9 g/kg DM for the beef diets and 59 ± 6.9 g/kg DM for the dairy diets, although the greatest lignin concentrations were 95 g/kg DM and 234 g/kg DM in the beef and dairy diets, respectively. Thus, in the current experiment, dietary lignin concentrations in both grape marc diets were much greater than the mean concentrations in both the beef and dairy diets reported by Ellis et al. [35], but substantially less than the highest concentration reported. The concentration of crude fat was numerically greater in the RGM than in the WGM and this resulted in the fat concentration of the total diet being 73 g/kg DM in the diet of cows offered the RGM compared to 64 g/kg DM in the diet of the cows offered the WGM. These dietary fat concentrations are greater than fat concentrations reported for beef diets (33 ± 1.7 g/kg DM) and dairy diets (37 ± 1.3 g/kg DM) [35]. The difference in CT and TAB-HCl for each grape marc, along with the LEM, is evidence of the processing differences between the production of RGM versus WGM as red grape marc necessarily undergoes additional oxidation during winemaking.

Cows fed grape marc instead of perennial ryegrass had smaller yields of milk, milk fat and milk protein, leading us to accept our first hypothesis. Yield of lactose and ECM were also reduced. This finding is similar to previous findings when grape marc was fed instead of alfalfa hay [14]. Reduced milk production in cows fed the grape marc instead of pasture was expected because both grape marcs had relatively low nutritional value as judged by their low ME because of their high concentrations of lignin, and low total digestible nutrients.

The finding of approximately 3 kg/d reduced ECM production when cows were fed diets containing approximately 4.5 kg DM/d of grape marc might prompt the question as to why farmers would feed grape marc to their cows. In Australia, in summer, when there is usually a shortage of available pasture, the choice is generally not between feeding more or less pasture, but between feeding some pasture or feeding some pasture and also a feed supplement such as grape marc. If cows in our CON treatment had been fed 9.5 kg DM of pasture instead of 13.3 kg DM of pasture, we would expect that their ECM yields would have been approximately 8 kg/d lower than measured, and approximately 5 kg/d lower than obtained from cows offered the RGM and WGM diets. Thus, the results of this experiment demonstrate how farmers make the most out of available resources, even when a resource such as grape marc, has relatively low nutritional value.

Cows fed either the RGM or the WGM diets had similar yields of milk, milk fat and milk protein, leading us to accept our second hypothesis. The similarity in yields of milk, ECM, milk fat, milk protein and milk lactose reflects the similar intakes of ME, and total digestible nutrients of the RGM and WGM diets. The concentration of milk fat in cows offered the RGM diet was less than the concentrations of milk fat in milk from cows offered the WGM diet. This observation may be a reflection of the greater fat intake by cows offered the RGM diet, as diets containing high (i.e., >6%) fat concentrations have been associated with reduced fibre digestion and reduced milk fat concentrations [36,37].

In the current experiment, we measured a methane yield of 20.6 g/kg DMI for the perennial ryegrass based CON diet. The magnitude of this methane yield is similar to the yield of 20.7 g methane/kg DMI reported by Charmley et al. [38] in a survey of methane yields in forage fed cattle in Australia.

Including either red or white grape marc in the pasture-based diet reduced methane emissions to an equal extent, approximately 15%, leading us to accept our third hypothesis. Our observed 15% decrease in methane emission and methane yield is consistent with findings of a 20% reduction in methane emission and methane yield in an experiment in which red grape marc was fed to dairy cows fed a basal diet of alfalfa hay [14].

The major components in grape marc that are likely to, or at least possibly contribute to the reduced production of methane include ash, fat, lignin, and CT and their potential roles in methane mitigation have been discussed by Moate et al. [14]. Furthermore, as the concentrations of these methane mitigating components differed independently in the red and white grape marcs used in this experiment, it is not possible to make conclusive statements as to the relative contribution of each of these components. However, based on information from the scientific literature, we can consider the likely effects of these components.

Ash is not fermented in the rumen and therefore does not contribute to the ruminal production of methane. In this experiment, the intake of ash was numerically less for the grape marc diets compared to the CON diet, and so ash concentration does not explain the reduced methane production on the grape marc diets.

Fat in temperate grass species, including perennial ryegrass, is mostly composed of mono-galactosyl diglyceride, which contains approximately 65% non-esterified fatty acids (NEFAs), 25% galactose and 10% glycerol [39]. When plant fat from temperate grasses undergoes lipolysis in the rumen, the resulting galactose and glycerol are readily fermented in the rumen, but the NEFAs do not undergo fermentation [27]. The above issues underpin the definition of PFOM that is provided in the materials and methods section. As well as not being fermented within the rumen, it is also believed that NEFAs coat the surface of feed particles within the rumen [40], and we postulate that this may be a mechanism by which dietary fat could inhibit ruminal fermentation and hence the production of methane. Moate et al. [3] showed that for each increase of 10 g/kg DM in dietary lipid concentration, enteric methane emissions are reduced by 0.79 g CH_4_/kg DMI. In this experiment, the total fat concentrations in the CON, RGM and WGM diets were 53, 73 and 65 g/kg DM, respectively. Thus, using the coefficient reported by Moate et al. [3], the higher fat concentrations in the grape marc diets can explain 1.58 g/kg DM or 49% of the reduction in methane yield for the RGM diet and 0.95 g/kg DM or 29% of the reduction in methane yield for the WGM diet.

Lignin is either not fermented or only very poorly fermented in the rumen [41] and therefore can be expected to not contribute to the ruminal production of methane. Furthermore, lignin may covalently bond to carbohydrates thus causing steric hindrance to carbohydrate-degrading enzymes resulting in reduced fermentation of carbohydrates [42], and hence providing an additional potential mechanism to explain how lignin might reduce ruminal production of methane. In this experiment, there was no direct evidence of reduced ruminal fermentation as total VFA concentrations in ruminal fluid were similar for all diets. This finding is surprising given that in comparison to the CON diet, both grape marc diets had significantly reduced ME intake. We surmise that the similarity of total VFA concentrations across all dietary treatments may reflect the fact that total VFA concentrations are unreliable estimators of treatment effects on ruminal fermentation in vivo [43]. However, we note that the reduced production of ECM for both grape marc diets compared to the CON diet, is indirect evidence that the grape marc diets were probably associated with reduced ruminal fermentation and possibly reduced methane production. If we assume that in this experiment, lignin was not fermented in the rumen and did not produce methane, then we can estimate that the 17.6 kg DM constituting the non-lignin part of the control diet, was responsible for 383 g CH_4_ and that this non-lignin part of the control diet has a methane yield of approximately 21.8 g CH_4_/kg of non-lignin DM. Compared to the CON diet, the approximately 1.7 kg DM of additional lignin in the RGM diet prevented (1.7 × 21.8) approximately 37 g CH_4_/d from being produced, and can explain approximately 2.0 g CH_4_/kg DMI or 63% of the reduction in methane yield. Compared to the CON diet, the 1.57 kg of additional lignin in the WGM diet prevented approximately 34 g CH_4_/d from being produced and may explain approximately 1.8 g CH_4_/kg DMI or 60% of the reduction in methane yield from the WGM diet. The combined effects of fat and lignin can account for 112% of the reduction in methane yield due to the RGM diet and 89% of the reduction in methane yield due to the WGM diet.

Condensed tannins from grape seeds have been shown to have a direct inhibitory effect on methanogenesis in vitro [44]. However, findings from experiments conducted in vitro do not always relate to what happens in vivo [34,45]. In the current experiment, the apparent lack of a major effect of grape tannins on methanogenesis is not surprising as there is great diversity in the total concentration and the different types of tannins present in different types and sources of grape marc [15,16,17]. Furthermore, not all types of tannins inhibit methanogenesis. An experiment with growing beef cattle found that when tannin from the Quebracho tree (*Schinopsis lorentzi* L) constituted approximately 20 g/kg DM of dietary DMI, there was no effect on enteric methane emissions [46]. By comparison, it can be calculated that in the current experiment, the concentrations of total CT were approximately 1.6 g/kg in the RGM diet and 13.5 g/kg DM in the WGM diet. Alternatively, the tannin concentration as determined by TAB-HCl yields a diet concentration of 11.4 and 25.9 g/kg of DMI for the RGM and WGM diets, respectively, highlighting the difficulty in determining the impact of tannin with varying tannin structures and nature (how tightly bound to feed protein and carbohydrate), and using different analytical techniques. The fact that the reduction in methane emissions was similar for the RGM and WGM diets despite a substantial difference in the concentration of CT in these two grape marcs supports the contention that the CT from grape marc is not particularly potent at inhibiting methanogenesis in comparison to large changes in lignin and ME concentrations. It has been reported that the tannin in red wines interacts or binds with dietary lipids [47]. We surmise it is possible that during the crimping and steam distillation process used to produce the grape marc, some of the grape tannins may have bound to fats released from the grape seeds. If this did occur, it is possible that when ingested by cattle, the tannins in grape marc may reduce the anti-methanogenic effects of the fats in the grape marc.

Our estimates of methane per kilogram of PFOM were similar between diets. This suggests that the reduction in methane emissions and methane yield (g/kg DMI) due to feeding grape marc can mostly be explained by the differences in unfermentable material between the CON and grape marc diets. In our experiment, the magnitudes of PFOM in the different diets were greatly dependent on lignin concentrations of dietary ingredients. Many published articles dealing with the effects of different diets on methane emissions and yields have not reported lignin concentration in diets. Therefore, it is not surprising that recent reviews on dietary factors that influence methane emissions and methane yields have generally not mentioned lignin [48,49]. Furthermore, with the exception of the equations presented by Ellis et al. [35,50], lignin has generally not been included in equations that have attempted to predict methane yields on the basis of dietary composition [51]. The current research highlights that lignin may have a major impact on methane emissions and yields and that this impact should be considered when developing equations to predict enteric methane emissions.

Milk fatty acid concentrations were affected by replacement of a portion of pasture with grape marc. Feeding of either RGM or WGM as a substitute for pasture resulted in reduced percentages of the C6 to C16 individual fatty acids, reduced total de novo fatty acids, and increased percentages of C18:0, C18:1 *trans*-9, C18:1 *trans*-10, C18:2n6 *cis*, total C18 and total polyunsaturated fatty acid. These findings are consistent with previous findings with red grape marc [14]. The reduced percentages of the de novo fatty acids are consistent with the fact that both grape marc diets contained reduced amounts of fermentable carbohydrate, the precursor of de novo fatty acids [52]. The feeding of diets containing grape marc was associated with enhanced percentages of the pre-formed fatty acids (i.e., fatty acids with more than 18 carbon atoms), reflecting that both grape marcs had a high fat concentration containing substantial amounts of C18:1 *cis*-9 and C18:2 n6. Generally, both grape marcs had similar effects on the percentages of the specific fatty acids in milk fat. However, the RGM had a greater depressive effect on the percentages of individual de novo fatty acids and a greater enhancing effect on the percentages of the pre-formed fatty acids in comparison to the WGM. As in previous research with red grape marc, in this experiment, in comparison to the CON diet, the RGM diet resulted in enhanced percentages of CLA, total mono-unsaturated fatty acids and total poly-unsaturated fatty acids, which are all regarded as being beneficial for human health [53,54]. Furthermore, in comparison to the WGM diet, the RGM diet also resulted in enhanced concentrations of these fatty acids indicating that red grape marc is superior to white grape marc for modifying the fatty acid composition of milk fat.

Changes in proportions of ruminal VFAs are often associated with changes in methane production [55]. Diets rich in fermentable carbohydrates, especially starch, result in increased production of propionate in the rumen and a decrease in the ratio of acetate to propionate [56]. Production of propionate acts as a sink for metabolic hydrogen and therefore competes with methane production [41]. Thus, diets that reduce acetate to propionate ratio are generally associated with reduced methane yield. In this regard, Carulla et al. [57] supplemented the diet of sheep with a tannin extract from black wattle and found that both ruminal acetate to propionate ratio and methane emissions were decreased. However, contrary to this finding, Beauchemin et al. [46] found that when a diet containing CT extract from quebracho trees was fed to cattle, the acetate to propionate ratio in ruminal fluid was decreased, but there was no effect on methane emissions. In considering the mechanism by which the feeding of grape marc may inhibit enteric methane production, it is of interest that in the current experiment, compared to the control diet, the two grape marc diets did not reduce the acetate to propionate ratio. This observation provides further evidence that in this experiment, the reduction in methane production by grape marcs did not involve the production of metabolic hydrogen or a direct inhibition of methanogenesis.

Elevated dietary concentrations of condensed tannins and fats have sometimes been reported to reduce the numbers of ciliated protozoa in ruminal fluid, and these protozoa are known to harbor methanogenic microbes [58,59,60]. In the current experiment, as in our previous experiment with diets containing red grape marc [14], the RGM and WGM diets had no effect on the counts of the two most common genera of ciliated protozoa: *Entodinium* and *Epidinium*, or on counts of total protozoa in ruminal fluid. Thus, in this experiment, the anti-methanogenic effect of the grape marc diets was not via an effect on ruminal protozoa.

## 5. Conclusions

Partial replacement of freshly harvested, single-chop perennial ryegrass with either RGM or WGM in the diet of dairy cows resulted in reduced yields of milk, ECM, milk fat, milk protein and milk lactose and reduced the methane emissions and methane yields (g/kg DM) with no differences between the RGM and WGM for these parameters. Compared to perennial ryegrass, both the RGM and the WGM had greater concentrations of crude fat and lignin which are either not fermented or poorly fermented in the rumen. We conclude that most of the effects of both grape marcs on milk production can be attributed to reduced ME intake. The effects of grape marcs on methane variables can be attributed to the greater concentrations of crude fat and lignin in the grape marcs and that grape tannins may have had only a minor effect on methane emissions.

## Figures and Tables

**Table 1:** Composition of dietary ingredients (g/kg dry matter, DM, unless otherwise stated).

Parameter	Pasture	Cold-Pressed Canola	Corn	Red Grape Marc	White Grape Marc
DM (g/kg)	137	902	861	436	415
Crude protein	218	366	84	132	122
Soluble protein (% of crude protein)	43	24	15	16	17
Acid detergent fibre	338	255	51	581	431
Neutral detergent fibre	523	335	108	615	499
Lignin	51	95	11	426	412
Non-fibre carbohydrate	139	194	784	107	249
Starch	17	11	737	11	28
Simple sugars	83	135	28	34	21
Ash	127	63	11	74	80
Total digestible nutrients	600	760	860	440	490
PFOM ^1^	788	746	955	377	417
GE (MJ/kg DM)	18.6	20.1	18.5	20.6	19.9
ME (MJ/kg DM)	9.9	13.8	13.9	6.7	7.5
Sodium	2.6	0.42	0.04	0.05	0.1
Potassium	34.1	11.6	2.6	19.5	18.2
Calcium	5.6	5.9	0.1	7.7	5.6
Magnesium	2.1	5.1	0.7	1.1	1.2
Phosphorus	3.9	9.2	2.2	2.8	3.0
Chloride	9.3	0.80	0.8	0.6	0.9
Sulphur	3.4	6.1	1.1	1.7	1.5
Crude fat	52	107	26	136	102
Fatty acids as % of total fatty acids					
C16:0	12.1	5.8	13.3	9.1	9.3
C16:1	2.1	0.5	ND	0.2	0.2
C18:0	1.2	1.8	2.4	4.1	4.1
C18:1 *cis*-9	1.6	58.2	30.8	15.1	15.3
C18:1 *cis*-11	ND	6.0	0.5	0.7	0.7
C18:2 n6	11.2	20.0	51.6	68.9	68.1
C18:3 n3	70.9	6.8	1.0	0.3	1.2
Other	0.9	0.9	0.5	0.9	1.1

^1^ PFOM = potentially fermentable organic matter; GE = gross energy; ME = metabolizable energy.

**Table 2:** Concentrations of tannins in red grape marc (Red) and white grape marc (White).

Tannin Analysis	Red	White
Phloroglucinolysis method		
CT ^1^ (g/kg DM)	6.9	60.3
LEM ^2^ (g/kg DM)	7.7	22.5
mDP ^3^ (molar ratio)	12.4	9.6
Cis/trans ^4^ (molar ratio)	10.1	9.3
Tri-OH ^5^ (% of CT)	20.1	7.3
Galloylation ^6^ (% of CT)	8.8	12.9
HBT ^7^ (g/kg DM)	20.1	16.5
TAB-HCl ^8^ (g/kg DM)	48.7	115.9
WET ^9^ (g/kg DM)	0.92	1.01

^1^ CT = total condensed tannins analysed by the phloroglucinolysis method; ^2^ LEM = late eluting material analysed by the phloroglucinolysis method; ^3^ mDP = mean degree of polymerization from the ratio of total to terminal sub units; ^4^ *Cis*/*trans* = ratio of the 2,3-*cis*-based to 2,3-*trans* based subunits; ^5^ Tri-OH = percentage of subunits with trihydroxylated B-rings; ^6^ Galloylation = percentage galloylation based on the occurrence of epicatechin-O-3-gallate terminal and extension subunits; ^7^ HBT = highly bound tannin analysed by an acetone containing Porter’s assay on the whole marc and on the fibres remaining after phloroglucinolysis [29]; ^8^ TAB-HCL = tannin analysed by the acetone-butanol-HCl method; ^9^ WET = water extractable tannin as analysed by the methyl cellulose precipitation assay.

**Table 3:** Selected nutritional characteristics (g/kg DM unless otherwise specified) of the feed eaten by the cows.

Parameter	CON ^1^	RGM	WGM
Crude protein	210	190	188
Acid detergent fibre	279	341	304
Neutral detergent fibre	430	459	430
Starch	133	123	129
Crude fat	53	73	65
Lignin	49	137	132
Total digestible nutrients	653	611	624
Condensed tannin	-	1.6	13.5
ME ^2^ (MJ/kg DM)	10.9	10.0	10.2
Ash	11.1	9.9	10.1

^1^ CON = control diet; RGM = red grape marc diet; WGM = white grape marc diet; ^2^ ME = metabolizable energy.

**Table 4:** Influence of diet on feed and nutrient intake (kg DM/d unless specified otherwise).

Variate	Diet ^1^			SEM ^2^	Effect *p*-Value	
	CON	RGM	WGM		C v G ^3^	R v W ^4^
No. of cows	12	10	10			
Pasture	13.3	9.5	9.4			
Cold-pressed canola	2.0	1.8	1.8			
Corn	3.0	2.8	2.9			
Mineral mix	0.20	0.20	0.20			
Red grape marc	0	4.5	0			
White grape marc	0	0	4.3			
Total	18.5	18.8	18.6	0.29	0.637	0.578
Nutrient intake						
Metabolizable energy (MJ/d)	200	189	190	3.2	0.008	0.982
Crude protein	3.86	3.57	3.49	0.059	0.001	0.289
Acid detergent fibre	5.14	6.43	5.65	0.107	0.021	0.003
Neutral detergent fibre	7.92	8.65	7.99	0.136	0.021	0.003
Fat	0.98	1.37	1.20	0.026	0.001	0.002
Lignin	0.90	2.59	2.47	0.068	0.001	0.212
Total digestible nutrients	12.0	11.5	11.6	0.20	0.047	0.956
Condensed tannin (g/d)	0	31	261	8.9	0.001	0.001
PFOM ^5^	14.8	13.3	13.3	0.21	0.001	0.929
Ash	2.04	1.87	1.87	0.031	0.001	0.935

^1^ Diets: CON = control diet, RGM = red grape marc diet, WGM = white grape marc diet; ^2^ SEM = standard error of mean; ^3^ *p*-value to test contrast between CON and grape marc; ^4^ *p*-value to test contrast between red grape marc and white grape marc; ^5^ PFOM = potentially fermentable organic matter.

**Table 5:** Influence of diet on milk production, milk composition and change in body weight.

Variate	Diet ^1^			SEM ^2^	Effect *p*-Value	
	CON	RGM	WGM		C v G ^3^	R v W ^4^
No. of cows	12	10	10			
Milk production (kg/d)						
Milk yield	29.1	26.9	26.0	0.44	0.001	0.225
Energy corrected milk	29.4	26.1	26.5	0.44	0.001	0.512
Fat	1.22	1.05	1.1 1	0.026	0.001	0.092
Protein	0.90	0.81	0.79	0.013	0.001	0.543
Lactose	1.48	1.34	1.32	0.024	0.001	0.478
Fat plus protein	2.12	1.85	1.91	0.033	0.001	0.296
Milk composition (%)						
Fat	4.23	3.96	4.30	0.081	0.342	0.014
Protein	3.11	3.01	3.06	0.030	0.040	0.357
Lactose	5.09	5.05	5.03	0.030	0.148	0.723
log10 SCC ^5^	1.65	2.02	1.68	0.180	0.395	0.239
Change in BW (kg/d)	0.78	0.30	0.67	0.149	0.102	0.082

^1^ Diets: CON = control diet, RGM = red grape marc diet, WGM = white grape marc diet; ^2^ SEM = standard error of mean ^3^ *p*-value to test contrast between CON and grape marc; ^4^ *p*-value to test contrast between red grape marc and white grape marc; ^5^ SSC = somatic cell count in cells/µL.

**Table 6:** Fatty acid composition of milk fat (g/100 g of fatty acid) from cows fed a control diet, a diet containing red grape marc or a diet containing white grape marc.

Fatty Acid	Diet ^1^			SEM ^2^	Effect *p*-Value	
	CON	RGM	WGM		C v G ^3^	R v W ^4^
Number of cows	12	10	10			
C4:0	4.18	4.42	4.32	0.075	0.055	0.424
C6:0	2.30	2.02	2.17	0.056	0.007	0.115
C8:0	1.22	0.96	1.05	0.042	0.001	0.185
C10:0	2.59	1.84	2.06	0.119	0.001	0.254
C10:1	0.21	0.13	0.16	0.008	0.001	0.019
C12:0	2.71	1.88	2.11	0.118	0.001	0.210
C14:0	10.93	8.21	9.21	0.354	0.001	0.083
C14:1	0.58	0.36	0.45	0.026	0.001	0.035
Iso C15:0	0.35	0.27	0.29	0.012	0.001	0.212
Anteiso C15:0	0.18	0.13	0.14	0.005	0.001	0.246
C15:0	0.77	0.61	0.63	0.021	0.001	0.465
C16:0	29.17	23.16	27.07	0.668	0.001	0.001
C17:0	0.40	0.37	0.36	0.010	0.047	0.617
C17:1	0.14	0.11	0.12	0.013	0.169	0.722
C18:0	13.95	18.07	17.22	0.456	0.001	0.246
C18:1 **trans*-*9	0.31	0.63	0.41	0.024	0.001	0.001
C18:1 **trans*-*10	0.27	0.67	0.40	0.029	0.001	0.001
C18:1 **trans*-*11	1.60	2.63	1.57	0.122	0.004	0.001
C18:1 *cis-*9	21.23	22.73	21.66	0.646	0.250	0.298
C18:1 *cis-*11	0.33	0.40	0.35	0.015	0.018	0.055
C18:2n6 *cis*	1.09	3.85	2.97	0.124	0.001	0.001
CLA ^5^	0.53	0.85	0.51	0.031	0.001	0.001
C18:3n3	0.60	0.56	0.54	0.015	0.013	0.494
C20:0	0.10	0.13	0.12	0.008	0.016	0.305
Total C18	39.92	50.38	45.64	1.118	0.001	0.012
Total *de novo* ^6^	41.31	32.94	36.75	0.891	0.001	0.012
Total saturated	40.11	39.31	40.01	0.435	0.421	0.316
Total unsaturated	59.89	60.69	59.99	0.426	0.579	0.598
mono-unsaturated	25.85	28.49	26.16	0.680	0.100	0.038
poly-unsaturated	1.70	4.41	3.51	0.130	0.001	0.001

^1^ Diets: CON = control diet, RGM = red grape marc diet, WGM = white grape marc diet; ^2^ SEM = standard error of mean ^3^ *p*-value to test contrast between CON and grape marc; ^4^ *p*-value to test contrast between red grape marc and white grape marc; ^5^ CLA = Conjugated linoleic acid; ^6^ total de novo = sum (C4.0 to C15:0) + 0.5 × (C16:0).

**Table 7:** Effect of feeding diets containing grape marc on methane emissions from dairy cows.

Variate	Diet ^1^			SEM ^2^	Effect *p*-Value	
	CON	RGM	WGM		C v G ^3^	R v W ^4^
Number of cows	11	10	10	-		
Total DMI ^5^ (kg/d)	18.4	18.8	18.6	0.29	0.425	0.578
Methane emission (g/d)	383	326	326	12.9	0.001	0.930
Methane intensity (g/kg ECM ^6^)	13.3	12.8	12.5	0.47	0.206	0.666
Methane yield (g/kg DMI)	20.6	17.4	17.4	0.68	0.001	0.925
Methane yield (g/kg PFOM ^7^)	26.5	25.1	25.1	0.79	0.143	0.991
Methane (% GEI ^8^)	6.24	5.06	5.18	0.179	0.001	0.650

^1^ Diets: CON = control diet, RGM = red grape marc diet, WGM = white grape marc diet; ^2^ SEM = standard error of mean ^3^ *p*-value to test contrast between CON and grape marc; ^4^ *p*-value to test contrast between red grape marc and white grape marc; ^5^ DMI = dry matter intake, ^6^ ECM = energy corrected milk (see text for calculation), ^7^ PFOM = potentially fermentable organic matter, ^8^ GEI = gross energy intake.

**Table 8:** Influence of diet on concentrations in rumen fluid of ammonia, total volatile fatty acids (VFAs), individual VFAs and rumen protozoa.

Variate	Diet ^1^			SEM ^2^	Effect *p*-Value	
	CON	RGM	WGM		C v G ^3^	R v W ^4^
Number of cows	12	10	10			
pH	6.77	6.78	6.81	0.059	0.712	0.787
NH_3_ (mg/L)	280	249	224	12.6	0.007	0.163
Ruminal fluid total VFA ^5^(mM)	85.9	85.9	83.4	2.60	0.686	0.504
Individual VFA (mM%)						
Acetic	69.0	69.1	71.0	0.402	0.028	0.003
Propionic	17.7	17.2	15.5	0.34	0.003	0.002
Iso-Butyric	1.30	1.24	1.13	0.033	0.006	0.025
n-Butyric	9.07	9.66	9.38	0.244	0.121	0.425
Iso-Valeric	1.84	1.66	1.70	0.069	0.063	0.666
n-Valeric	1.02	0.94	0.92	0.035	0.044	0.560
Caproic	0.11	0.14	0.36	0.035	0.002	0.001
Acetic:Propionic	3.90	4.04	4.59	0.103	0.002	0.001
Protozoa (Log_10_)						
*Entodinium* spp.	4.72	4.97	4.49	0.130	0.796	0.025
*Epidinium* spp.	2.37	2.81	2.47	0.200	0.226	0.217
Total	4.82	5.09	4.63	0.128	0.659	0.026

^1^ Diets: CON = control diet, RGM = red grape marc diet, WGM = white grape marc diet; ^2^ SEM = standard error of mean; ^3^ *p*-value to test contrast between CON and grape marc; ^4^ *p*-value to test contrast between red grape marc and white grape marc;^5^ VFA = volatile fatty acid.
